# 

**Published:** 1896-03-28

**Authors:** 


### CONTENTS.

The Discontent in the British Medical Association - 423

The Coming of Easter - 424

On Aneurisms. By A. Pearce Gould, F.R.C.S. - 425

The Care of the Sick in Prisons - 426

Medical Progress and Hospital Clinics—

The Treatment of Acquired Flat-foot. By A. H. Tubby, M.S.Lond., F.R.C.S.Eng. - 429

Progress in Surgery.—Surgery of the Vermiform Appendix - 431

Periscope of Dermatology - 432

New Appliances and Things Medical - 434

Annotations.—Dr. Jameson and South African Medicine—Traumatic Neurasthenia—Deaf Mutes and the School Board - 427

Notes and News - 428

The Institutional Workshop—

Hospital Expenditure: The Commissariat. XIII.—The Cost of the Diets - 435

Hospital Construction - 436

Hospital Meetings - 437

Editor's Letter-Box - 439

The Book World of Medicine and Science - 439

### EXTRA SUPPLEMENT.—THE NURSING MIRROR.

News from the Nursing World.—Royal Visit to Lancaster—Children's Hospital, Great Ormond Street—Honour where Honour is Due—Wanted, good Fever Nurses—Premature Promotion—York Home for Nurses—Small pox at Gloucester—A Wise Proposal Overruled—A Medical Woman at Aberdeen—Bury Dispensary Hospital—Short Items - ccxxvii

Private Nursing. IV.—Private Nurses—Surgical Cases - ccxxix

Burdett's Official Nursing Directory - ccxxix

On Certain Aspects of the Nursing Question as Seen in England and Germany. IV.—The Hebammen Schule ccxxx

Royal British Nurses' Association - ccxxx

Dress and Uniforms - ccxxxi

Training in New South Wales - ccxxxii

A Book and its Story - ccxxxiii

The Misuse of a Nurse's Uniform - ccxxxiv

Everybody's Opinion - ccxxxiv

News from Melbourne - ccxxxv

Practical Points - ccxxxv

Appointments - ccxxxv

Notes and Queries - ccxxxv

The Book World for Women and Nurses - ccxxxvi

For Reading to the Sick - ccxxxvi

